# Prediction of clinical benefit from androgen deprivation therapy in salivary duct carcinoma patients

**DOI:** 10.1002/ijc.32795

**Published:** 2019-12-12

**Authors:** Wim van Boxtel, Gerald W. Verhaegh, Ilse A. van Engen‐van Grunsven, Dianne van Strijp, Leonie I. Kroeze, Marjolein J. Ligtenberg, Hans B. van Zon, Yara Hendriksen, Diederick Keizer, Anja van de Stolpe, Jack A. Schalken, Carla M. van Herpen

**Affiliations:** ^1^ Department of Medical Oncology Radboud University Medical Center Nijmegen The Netherlands; ^2^ Department of Urology Radboud University Medical Center Nijmegen The Netherlands; ^3^ Department of Pathology Radboud University Medical Center Nijmegen The Netherlands; ^4^ Philips Research Eindhoven The Netherlands; ^5^ Department of Human Genetics Radboud University Medical Center Nijmegen The Netherlands; ^6^ Molecular Pathway Diagnostics Philips Healthworks Eindhoven The Netherlands

**Keywords:** salivary gland neoplasms, salivary duct carcinoma, androgen receptor antagonists, drug resistance, neoplasm, computational biology

## Abstract

Androgen deprivation therapy (ADT) is first‐line palliative treatment in androgen receptor‐positive (AR+) salivary duct carcinoma (SDC), and response rates are 17.6–50.0%. We investigated potential primary ADT resistance mechanisms for their predictive value of clinical benefit from ADT in a cohort of recurrent/metastatic SDC patients receiving palliative ADT (*n* = 30). We examined mRNA expression of androgen receptor (AR), AR splice variant‐7, intratumoral androgen synthesis enzyme‐encoding genes *AKR1C3*, *CYP17A1*, *SRD5A1* and *SRD5A2*, AR protein expression, *ERBB2* (HER2) gene amplification and DNA mutations in driver genes. Furthermore, functional AR pathway activity was determined using a previously reported Bayesian model which infers pathway activity from AR target gene expression levels. *SRD5A1* expression levels and AR pathway activity scores were significantly higher in patients with clinical benefit from ADT compared to those without benefit. Survival analysis showed a trend toward a longer median progression‐free survival for patients with high *SRD5A1* expression levels and high AR pathway activity scores. The AR pathway activity analysis, and not *SRD5A1* expression, also showed a trend toward better disease‐free survival in an independent cohort of locally advanced SDC patients receiving adjuvant ADT (*n* = 14) after surgical tumor resection, and in most cases a neck dissection (13/14 patients) and postoperative radiotherapy (13/14 patients). In conclusion, we are the first to describe that AR pathway activity may predict clinical benefit from ADT in SDC patients, but validation in a prospective study is needed.

## Abstract 2

**What's new?**

Androgen deprivation therapy (ADT) is a leading treatment strategy in the palliative care of patients with androgen receptor (AR)‐positive salivary duct carcinoma (SDC). However, while as many as half of patients may respond to ADT, resistance frequently emerges, undermining its use. In this investigation of primary ADT resistance mechanisms, expression of the androgen synthesis enzyme‐encoding gene *SRD5A1* and functional activity of the AR pathway were found to predict clinical benefit from ADT in SDC patients. High AR pathway activity scores were further linked to improved disease‐free survival in SDC patients with locally advanced disease who received adjuvant ADT.

**Abbreviations**
ADT: androgen deprivation therapy
AKR1C3: aldo‐keto reductase family 1 member C3
AR: androgen receptor
ASCO: American Society of Clinical Oncology
CAP: College of American Pathologists
CI: confidence interval
CRPC: castration‐resistant prostate cancer
CYP17A1: cytochrome P450 17A1
DFS: disease‐free survival
FFPE: formalin‐fixed paraffin‐embedded
FISH: fluorescent *in situ* hybridization
H&E: hematoxylin and eosin
HPRT1: hypoxanthine phosphoribosyltransferase 1
IQR: interquartile range
LA: locally advanced
OS: overall survival
PFS: progression‐free survival
R/M: recurrent/metastatic
ROC: receiver operating characteristic
SDC: salivary duct carcinoma
smMIP: single‐molecule molecular inversion probe
SRD5A1/2: steroid 5 alpha‐reductase 1/2

## Introduction

Salivary duct carcinoma (SDC) is an aggressive subtype of salivary gland cancer, which is often androgen receptor (AR) positive (66.7–96.4%).^1^, ^2^, ^3^ Primary treatment consists of a tumor resection, most often in combination with a neck dissection and postoperative radiotherapy. Despite this extensive treatment, the 3‐year disease‐free survival (DFS) rate is only 27.7% in locally advanced patients.^4^ In patients with recurrent and/or metastatic (R/M) SDC, androgen deprivation therapy (ADT) is often used as first‐line palliative treatment. In retrospective studies, ADT has shown response rates of 17.6–50.0% and an OS of 17 months compared to 5 months in a best supportive care cohort.^5^, ^6^ A recent prospective phase 2 trial in Japan showed a response rate of 41.7%, median progression‐free survival (PFS) of 8.8 months and median OS of 30.5 months.^7^ Because of the efficacy of ADT in R/M SDC patients, we evaluated ADT as adjuvant treatment in 22 patients with locally advanced (LA) AR‐positive SDC. Multivariable Cox regression analysis showed a significantly improved DFS (hazard ratio 0.14, 95% CI 0.03–0.75, *p* = 0.022) and OS (hazard ratio 0.06, 95% CI 0.01–0.76, *p* = 0.030) compared to 111 controls who did not receive adjuvant ADT.^4^

Besides ADT, other treatment options are available for patients with R/M SDC. In the case of *ERBB2* (HER2) gene amplification (29.4–46.4%),^1^, ^2^ patients can be treated with docetaxel plus trastuzumab, showing an overall response rate of 70.2% and median PFS of 8.9 months.^8^ Double HER2 blockade with docetaxel–trastuzumab–pertuzumab or in second‐line with the antibody‐drug conjugate trastuzumab‐emtansine also showed promising results.^9^, ^10^, ^11^ Finally, the high frequency (61.3%) of oncogenic driver gene mutations offers personalized treatment options.^12^

Despite the efficacy of ADT in the palliative and adjuvant setting, ADT is only effective in a subgroup of patients and little is known about primary resistance mechanisms. Although AR expression, determined by immunohistochemistry, is a hallmark of SDC, intratumoral and intertumoral variation of AR expression is frequently observed.^13^ Therefore, variation in AR mRNA and protein levels may cause variable responses. Furthermore, AR‐V7, an AR splice variant that lacks the ligand‐binding domain and is constitutively active, may cause ADT resistance. In prostate cancer *AR‐V7* expression is 20‐fold higher in castration‐resistant prostate cancer (CRPC) compared to hormone‐naïve prostate cancer, though in SDC the presence of *AR‐V7* has also been shown in hormone‐naïve tumors.^14^, ^15^ Another ADT resistance mechanism described in CRPC is increased expression of genes involved in intratumoral androgen synthesis.^16^ Key enzymes involved in the conversion of androgen precursors, such as dehydroepiandrosterone into dihydrotestosterone are aldo‐keto reductase family 1 member C3 (*AKR1C3*), cytochrome P450 17A1 (*CYP17A1*), steroid 5 alpha‐reductase 1 *(SRD5A1)* and *SRD5A2*. Finally, a low‐active or inactive AR signal transduction pathway, in which androgen stimulation does not result in (full) AR transcriptional activity, may cause primary ADT resistance, simply because no effective AR signaling is present. AR pathway activity can be quantified by a recently developed and validated method, in which expression of AR target genes is measured and subsequently converted into a pathway activity score (ranging between 0 and 100) using a Bayesian computational model.^17^, ^18^ Besides, these AR‐related mechanisms, primary ADT resistance may be caused by activity of other tumor‐driving pathways, for instance induced by *ERBB2* gene amplification or other tumor‐driving gene mutations. The aim of our study was to assess these potential primary ADT resistance mechanisms in a cohort of R/M SDC patients receiving palliative ADT and a cohort of LA SDC patients receiving adjuvant ADT. For those factors that differed significantly between R/M SDC patients with and without clinical benefit from ADT, the optimal cut‐off value and survival differences were assessed. Subsequently, this cut‐off value was used to evaluate DFS differences in the LA cohort.

## Methods

### Patients

Clinicopathological characteristics and potential ADT resistance mechanisms were assessed in a cohort of R/M AR‐positive SDC patients receiving palliative ADT (*n* = 30) and a cohort of LA AR‐positive SDC patients receiving adjuvant ADT (*n* = 14) after surgical tumor resection, and in most cases a neck dissection (13/14 patients) and postoperative radiotherapy (13/14 patients). ADT consisted of bicalutamide or LHRH‐analog plus bicalutamide following shared decision making.^5^ Patients were treated in the Radboud university medical center, Nijmegen, the Netherlands, or received a second opinion in the Radboud university medical center and were treated under supervision elsewhere in the Netherlands.

Our study was approved by the local medical ethical committee (file code 2017‐3917). Clinical data were collected from the medical records. A no‐objection system was used for secondary use of human tissue and medical data in accordance with the code of conduct of the Federation of Dutch Medical Scientific Societies (Human tissue and medical research: Code of conduct for responsible use). For all patients who were alive at the start of our study, written informed consent was obtained.

### Tissue

Formalin‐fixed paraffin‐embedded (FFPE) tissue samples of the primary tumor prior to treatment were collected (*n* = 36). If primary tumor material was unavailable, tumor material of locoregional lymph node metastases or distant metastases prior to treatment was used (*n* = 5 and *n* = 3, respectively). Nonstained FFPE sections were cut and used for DNA/RNA isolation, AR immunohistochemistry (IHC) and *ERBB2* FISH. Before and after these sections, hematoxylin and eosin (H&E) stained sections were prepared for pathological confirmation of SDC and for tumor annotation. Median percentage of neoplastic cells in tissue sections used for further analysis was 70.0% (range 25.0–90.0%) in the R/M cohort and 70.0% (range 30.0–80.0%) in the LA cohort.

### RNA isolation

For RNA extraction, three 10 μm sections were used. RNA was extracted from the marked tumor regions according to the manufacturer's protocol (Siemens, VERSANT® Tissue Preparation Reagents kit). RNA was eluted in 100 μl buffer according to the instructions. After DNase treatment (Siemens, Munich, Germany, VERSANT® Tissue Preparation Reagents kit) RNA was stored at −80°C. RNA concentration was measured using the Qubit® RNA HS Assay Kit (Thermo Fisher, Waltham, MA) on a Qubit® Fluorometer.

### Reverse transcriptase and real‐time PCR analysis

One microgram of total DNase‐treated RNA was used for cDNA synthesis. Random‐primed cDNA was synthesized using SuperScript II reverse transcriptase (Invitrogen, Carlsbad, CA). Gene expression levels were determined by SYBR Green qPCR (Roche, Basel, Switzerland) using a LightCycler LC480 instrument (Roche). Relative gene expression levels of *AR*, *AR‐V7*, *AKR1C3*, *CYP17A1*, *SRD5A1* and *SRD5A2* were calculated using the ΔΔCt method using the hypoxanthine phosphoribosyltransferase 1 (*HPRT1)* gene for normalization. Primer sequences are listed in Supporting Information Table S1.

### AR immunohistochemical staining

Androgen receptor expression was determined using the AR specific polyclonal antibody N‐20 (Santa Cruz Biotechnology, Santa Cruz, CA). FFPE sections were pretreated with citrate (pH 6.0) for 10 min in a pretreatment module. Immunostaining was carried out using a 1:200 dilution of the primary antibody, and staining was performed according to the Powervision method by Immunologic. The AR staining was scored considering the staining intensity (0 = negative, 1 = weak, 2 = moderate, 3 = strong) and the percentage of positive nuclei (0 = <10%, 1 = 10–30%, 2 = 30–70%, 3= >70%). The final staining score was recorded as the sum of the staining intensity and the staining extent.^6^

### AR pathway analysis

To quantitatively assess the AR pathway activity a qPCR‐based AR pathway test was used, that was adapted from the previously described biologically validated AR pathway analysis method developed for Affymetrix U133Plus2.0 microarray.^17^, ^18^, ^19^ The qPCR AR pathway test was developed to allow the use of FFPE material and small sample inputs (Philips Molecular Pathway Diagnostics, Eindhoven, The Netherlands). For the qPCR‐based AR pathway test, the relevant AR target gene expression levels were measured on FFPE tissue‐derived total RNA using one‐step RT‐qPCR and a Bayesian pathway model was used to quantitatively assess pathway activity.^18^ For the target genes and reference genes, multiple diagnostic grade qPCR assays were developed according to standard procedures. A human AR‐positive prostate carcinoma cell line (LNCaP) with ground‐truth data with respect to AR pathway activity was used for calibration of the qPCR‐based Bayesian model, similar as described for the Affymetrix‐based model.^18^ Based on a probability score (*p*), the odds for AR pathway activity was provided by the Bayesian model (*p*/1 − *p*), and this was transformed into a (base 2) logarithmic scale and then normalized to scores ranging from 0 to 100, where 0 corresponds to the lowest and 100 corresponds to the highest odds in favor of an active AR pathway that a specific model can infer. For each analyzed sample, this normalized AR pathway activity score between 0 and 100 was calculated.

### HER2 assessment

HER2 status was determined by *ERBB2* fluorescent *in situ* hybridization (FISH) according to standard ISH protocol using the ERBB2 probe of Kreatech (KI‐10701, mapping 17q12). Scoring was performed according to breast cancer guidelines of the American Society of Clinical Oncology (ASCO) and the College of American Pathologists (CAP).^20^ In case of an inconclusive ISH results, HER2 IHC was determined using the polyclonal rabbit antihuman c‐erbB‐2 antibody of DAKO according to protocol.

### DNA isolation and mutation analysis

For all patients in the R/M cohort, oncogenic driver gene mutations were assessed using single‐molecule molecular inversion probes (smMIPs) analysis and Next‐Generation Sequencing.^21^ DNA from FFPE tissue specimens was isolated as described and a validated 29‐gene panel was used to detect driver mutations (Supporting Information Table S2).

### Statistical analysis

Clinical ADT response in the R/M cohort was classified into five categories according to RECIST criteria: complete response, partial response, stable disease longer than 6 months, stable disease shorter than 6 months and progressive disease.^22^ Subsequently, patients with a complete response, partial response or stable disease >6 months were classified as “clinical benefit” Gene expression levels and AR pathway activity scores in tumor tissue of patients with and without clinical benefit compared by a two‐tailed independent *t*‐test with equal variance assumed. Equal variance was confirmed by Levene's test for equality of variances. A log transformation, using the natural logarithms, was performed on gene expression data before *t*‐tests were conducted in order to satisfy the assumption of normal distribution. AR protein expression levels in tumor tissue of patients with and without clinical benefit were compared using the Mann–Whitney U test. HER2 status and the presence or the absence of a driver mutation in patients with and without clinical benefit were compared using Fisher's exact test. Values of *p* < 0.05 were considered statistically significant without correction for multiple testing, as these analyses are considered exploratory.

For those factors that differed significantly between patients with and without clinical benefit, receiver operating characteristic (ROC) curves were constructed to establish the optimal cut‐off value to predict clinical benefit. Subsequently, Kaplan–Meier survival curves were constructed for patients above and below the cut‐off value. The log‐rank test was used to compare survival data. Finally, Pearson correlations were calculated for *AR* and *AR‐V7* gene expression levels, and for *SRD5A1* gene expression levels and AR pathway activity scores. Analyses were performed using IBM SPSS version 25.0.

## Results

### Patient characteristics

In the R/M cohort, 30 patients (22 men, 8 women) who received palliative ADT were analyzed. Median age at the start of ADT was 62 years (range 36–79 years). Seven patients were treated with a LHRH‐analog plus bicalutamide, and 23 patients with bicalutamide monotherapy. Of these 30 patients, 5 had a partial response (16.7%), 4 had stable diseases for more than 6 months (13.3%), 3 had stable diseases for less than 6 months (10.0%) and 18 (60.0%) had progressive disease at first evaluation. Median FFPE tissue storage time was 49 months (range 7–195 months), and 23 samples were obtained from primary tumor, four from regional lymph nodes metastases and three from distant metastases. Further patient characteristics are listed in Table 1.

**Table 1:** Patient and tumor characteristics of the recurrent/metastatic cohort, sorted by the AR pathway activity score

Pt no.	Gender	Tumor tissue	Tumor %	Age tissue (mo.)	*AR*	*AR‐V7*	AR IHC	AR pathway activity	*AKR1C3*	*SRD5A1*	*SRD5A2*	HER2 status	Genetic alterations*	ADT	Clinical response	PFS (mo.)	OS (mo.)
1	F	T	70	46	0.278	0.005	5.0	33.1	0.072	0.637	0.001	Pos	None	LHRH + bica	PD	3	29+
2	F	N	60	45	0.030	0.000	4.0	33.9	0.712	0.865	0.000	Neg	TP53	Bica	PD	2	5
3	M	T	50	136	0.260	0.001	6.0	34.8	2.099	3.117	0.001	Pos	TP53	Bica	PD	3	9
4	M	T	60–70	133	0.345	0.004	5.0	36.1	0.061	1.741	0.008	Pos	TP53 and ERBB2	Bica	PD	2	12
5	F	T	70	73	1.476	0.015	6.0	36.2	0.148	0.953	0.001	Neg	PTEN and TP53	Bica	PD	3	7
6	M	T	25	50	1.097	0.008	5.0	38.9	0.075	0.933	0.003	Neg	PTEN and TP53	Bica	SD >6 mo.	6	12
7	F	N	60–70	15	1.522	0.129	6.0	41.3	0.017	1.212	0.000	Neg	None	LHRH + bica	PD	9	14+
8	M	T	80	80	0.009	0.000	4.0	42.7	0.023	0.669	0.001	Pos	TP53	Bica	PD	0	0
9	M	T	80	38	0.615	0.021	3.0	43.2	0.707	1.111	0.021	Pos	None	Bica	PD	2	34+
10	M	T	70	8	5.169	0.737	6.0	43.6	2.313	9.646	0.000	Neg	None	Bica	PD	2	3+
11	F	M (epidural)	30	7	0.063	0.001	0.0	43.7	0.323	0.674	0.001	Pos	HRAS and PIK3CA	LHRH + bica	PD	0	10
12	M	T	70	61	1.409	0.006	5.0	44.3	0.332	2.854	0.001	Neg	AKT1 and BRAF	Bica	SD >6 mo.	18	40+
13	M	T	70	66	1.251	0.006	6.0	45.3	0.093	3.622	0.000	Pos	TP53 and ERBB2	Bica	PR	27	56+
14	F	T	70	195	3.227	0.454	6.0	45.4	0.732	5.540	0.001	Neg	HRAS and PIK3CA	Bica	PD	1	13
15	F	T	60	131	0.414	0.009	2.0	45.6	0.521	0.927	0.000	Neg	None	LHRH + bica	PD	2	5
16	F	T	40	48	0.082	0.001	2.0	46.6	0.065	1.206	0.001	Neg	None	LHRH + bica	PD	1	25
17	M	T	70	27	0.605	0.006	6.0	46.7	0.461	0.919	0.000	Neg	None	Bica	SD <6 mo.	5	12+
18	M	T	70	47	9.105	0.309	6.0	47.9	1.385	8.000	0.000	Neg	BRAF	Bica	PR	6	40+
19	M	T	30	13	5.046	0.081	6.0	48.8	0.732	5.429	0.007	Neg	None	Bica	PD	2	2+
20	M	T	30	25	1.598	0.038	6.0	49.4	0.395	8.877	0.003	Neg	None	Bica	PD	3	17
21	M	M (liver)	80	91	–	–	6.0	50.4	–	–	–	Neg	TP53#	Bica	PD	1	7
22	M	T	70	88	1.057	0.005	5.0	51.9	0.174	2.099	0.003	Neg	TP53	Bica	PD	1	11
23	M	T	60	66	0.578	0.005	6.0	52.0	0.186	0.905	0.001	Pos	TP53	Bica	SD <6 mo.	5	33
24	M	N	80	7	4.666	0.196	5.5	52.7	0.993	0.827	0.013	Pos	TP53	Bica	PD	2	6+
25	M	T	70	43	0.567	0.003	5.0	53.2	0.082	2.060	0.019	Pos	None	LHRH + bica	SD >6 mo.	9	18+
26	M	T	90	66	4.522	0.207	6.0	54.5	0.282	4.710	0.010	Neg	PIK3CA	LHRH + bica	SD >6 mo.	8+	13+
27	M	T	70	20	1.094	0.186	6.0	56.7	0.056	3.216	0.003	Pos	None	Bica	PR	5	18+
28	M	T	70	39	2.438	0.262	6.0	57.6	0.448	2.636	0.027	Neg	HRAS and 2xPIK3CA	Bica	SD <6 mo.	1	5
29	M	M (skull base)	90	154	3.287	0.068	6.0	61.8	9.590	33.988	0.004	Neg	None	Bica	PR	10	20
30	M	N	60	101	2.746	0.069	6.0	65.6	1.148	21.472	0.008	Neg	HRAS and PIK3CA	Bica	PR	14.	44

Abbreviations: #, because of low DNA yield other mutations could have been missed; *, specific mutations are listed in Supporting Information Table S3; +, ongoing PFS/OS; ADT, androgen deprivation therapy; *AKR1C3*, aldo‐keto reductase family 1 member C3 gene expression; AR IHC, immunohistochemical androgen receptor expression; *AR*, androgen receptor gene expression; *AR‐V7*, androgen receptor splice variant 7 gene expression; bica, bicalutamide; F, female; LHRH, luteinizing hormone‐releasing hormone agonist; M, distant metastasis; M, male; mo., months; N, lymph node metastasis in neck; OS, overall survival; PD, progressive disease; PFS, progression‐free survival; PR, partial response; SD, stable disease; *SRD5A*, steroid 5 alpha‐reductase gene expression; T, primary tumor.

In the adjuvant ADT treated LA cohort, 14 patients (12 men, 2 women) were analyzed. Median age at the start of ADT was 61 years (range 36–84 years). Two patients were treated with an LHRH‐analog plus bicalutamide and 12 patients with bicalutamide monotherapy. Median FFPE tissue storage time was 13.5 months (range 3–108 months), and 13 samples were obtained from primary tumors and 1 from a regional lymph node metastasis. Further patient characteristics are listed in Table 2.

**Table 2:** Patient and tumor characteristics of the locally advanced cohort, sorted by the AR pathway activity score

Patient no.	Gender	Tumor tissue	Tumor %	Age tissue (mo.)	*AR*	*AR‐V7*	AR IHC	AR pathway activity	*AKR1C3*	*SRD5A1*	*SRD5A2*	HER2 status	ADT	DFS (mo.)	OS (mo.)
31	M	T	70	18	1.187	0.042	6.0	37.9	0.121	0.883	0.003	Neg	Bica	18+	18+
32	F	T	60	6	4.045	0.812	6.0	42.9	0.351	3.364	0.005	Neg	LHRH + bica	5+	5+
33	M	T	40	108	1.014	0.045	6.0	44.4	0.007	0.164	0.002	Neg	Bica	17	52
34	M	T	70	12	0.886	0.006	6.0	45.3	0.883	1.050	0.028	Pos	Bica	10+	10+
35	F	T	80	3	4.441	0.237	5.5	46.0	0.829	7.062	0.046	–	LHRH + bica	2+	2+
36	M	T	70	3	4.056	0.162	4.5	48.4	0.829	1.117	0.003	–	Bica	3+	3+
37	M	T	30	13	5.046	0.081	6.0	48.8	0.732	5.429	0.007	Neg	Bica	6	8+
38	M	N	60	36	1.253	0.022	6.0	49.8	0.521	5.464	0.011	Neg	Bica	12	22
39	M	T	80	23	1.366	0.067	6.0	53.3	0.183	10.724	0.068	Neg	Bica	22	34+
40	M	T	40	10	2.235	0.084	6.0	54.0	1.357	6.635	0.002	Neg	Bica	11+	11+
41	M	T	70	18	1.886	0.116	6.0	54.4	0.369	1.905	0.012	Pos	Bica	15+	15+
42	M	T	50	30	1.663	0.048	6.0	58.6	0.532	22.098	0.052	–	Bica	25+	25+
43	M	T	70	9	6.755	0.147	6.0	59.6	0.737	46.340	0.004	Neg	Bica	11+	11+
44	M	T	70	14	0.657	0.020	4.5	61.6	0.198	7.835	0.222	Pos	Bica	15+	15+

Abbreviations: +, ongoing DFS/OS; ADT, androgen deprivation therapy; *AKR1C3*, aldo‐keto reductase family 1 member C3 gene expression; AR IHC, immunohistochemical androgen receptor expression; *AR*, androgen receptor gene expression; *AR‐V7*, androgen receptor splice variant 7 gene expression; bica, bicalutamide; DFS, disease‐free survival; F, female; M, male; mo., months; N, lymph node metastasis in neck; OS, overall survival; *SRD5A*, steroid 5 alpha‐reductase gene expression; T, primary tumor.

### ADT resistance mechanisms

**Figure 1:**
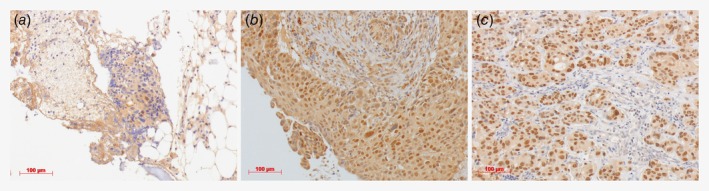
Androgen receptor (AR) immunohistochemical staining. Examples of a negative staining (score 0 + 0 = 0) in (*a*), moderate staining (score 2 + 2 = 4) in (*b*) and strong staining (score 3 + 3 = 6) in (*c*) are shown. The AR staining was scored as described in materials and methods. Images were taken at 200× magnification. [Color figure can be viewed at http://wileyonlinelibrary.com]

AR pathway activity scores and AR protein expression were determined in all patients in both cohorts. Insufficient RNA was available for gene expression analysis in one patient in the R/M cohort. Typical AR immunohistochemical staining expression patterns are shown in Figure 1. The difference in AR protein expression levels between patients with and without clinical benefit was not significant (median 6.0, interquartile range (IQR) 5.0–6.0 *vs*. 6.0, IQR 4.0–6.0, *p* = 0.29). Also, expression levels of *AR* (median 1.41 IQR 1.10–3.90 *vs*. 0.61, IQR 0.26–2.23, *p* = 0.054), *AR‐V7* (median 0.068, IQR 0.006–0.20 *vs*. 0.008, IQR 0.002–0.12, *p* = 0.49), *AKR1C3* (median 0.28, IQR 0.079–1.27 *vs*. 0.42, IQR 0.091–0.73, *p = 0*.*79)*, *SRD5A2* (median 0.003, IQR 0.0005–0.009 *vs*. 0.001, IQR 0.0003–0.006, *p* = 0.32), and *CYP17A1* (negative in all tumors) mRNA were not significantly different between patients with and without clinical benefit. However, *SRD5A1* mRNA expression levels were significantly higher (*p* = 0.008) in patients with clinical benefit (median 3.62, IQR 2.46–14.74) compared to those without (median 1.16, IQR 0.88–3.00). Moreover, the difference in AR pathway activity scores between patients with clinical benefit (median 53.2, IQR 44.8–59.3) and those without (median 45.4, IQR 38.7–49.9) was significant (*p* = 0.017). Box plots of ADT resistance mechanisms in patients with and without clinical benefit are shown in Supporting Information Figure S1. Although *AR‐V7* expression levels were not significantly different in both groups, 41 of 43 evaluated patients in both cohorts (95.3%) had detectable *AR‐V7* levels, and a significant correlation between *AR* and *AR‐V7* expression levels was found (*R* ^2^ = 0.34, *p* < 0.001; Supporting Information Figure S2).

### Prediction of clinical benefit from ADT using the AR pathway activity score

**Figure 2:**
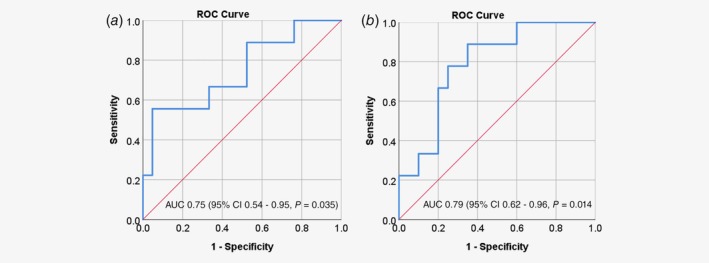
Receiver operating characteristic (ROC)‐curves describing the sensitivity and specificity to predict clinical benefit from androgen deprivation treatment in the R/M cohort. (*a*) ROC‐curve of androgen receptor pathway analysis. A cut‐off value of 52.9 was used for the subsequent survival analyses, which has a sensitivity of 0.556 and 1‐specificity of 0.048 in this cohort. (*b*) ROC‐curve of steroid 5 alpha‐reductase 1 (SRD5A1) gene expression levels. A cut‐off value of 2.75 was used, which has a sensitivity of 0.778 and one specificity of 0.250 in this cohort. AUC, area under the curve; CI, confidence interval. [Color figure can be viewed at http://wileyonlinelibrary.com]

**Figure 3:**
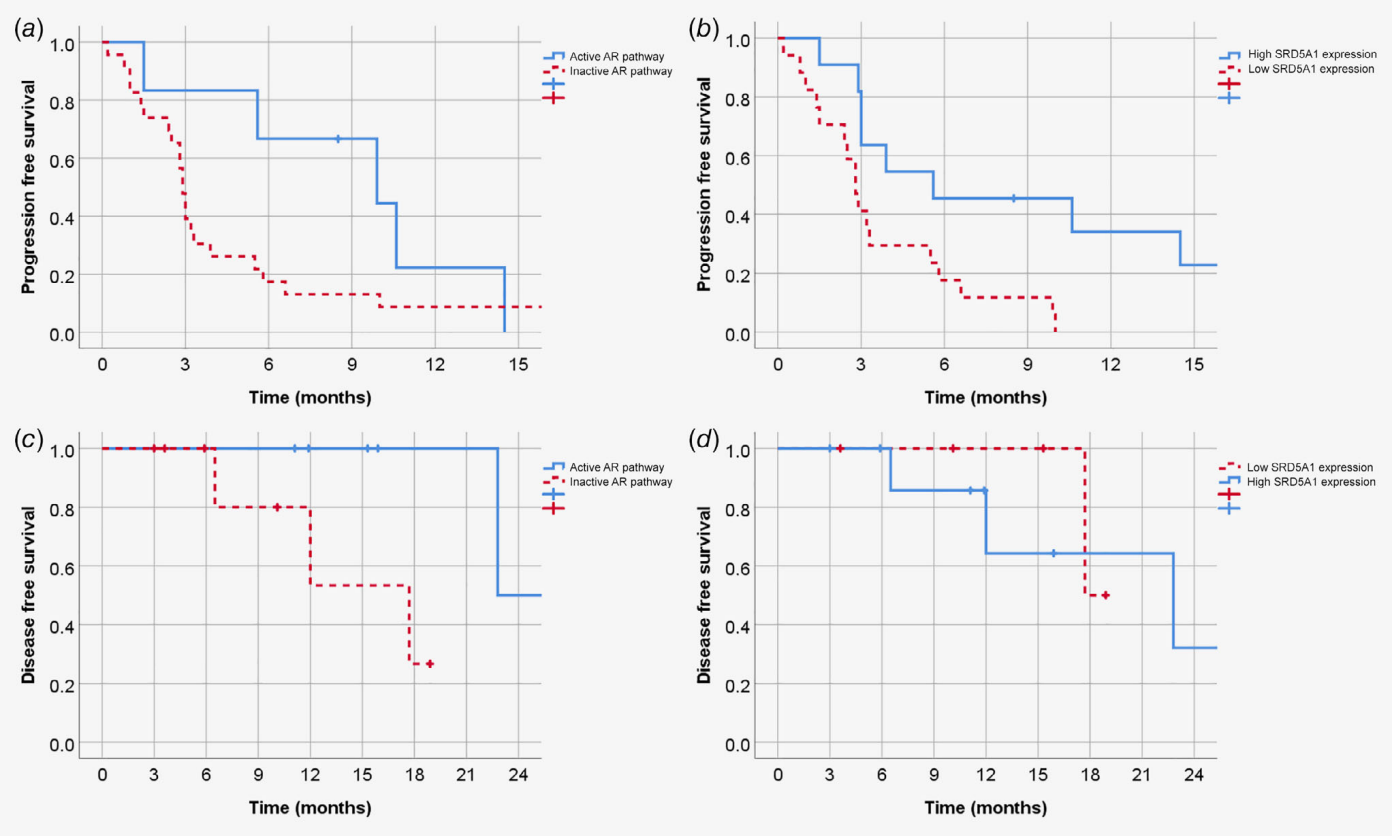
Kaplan–Meier survival curves. Progression‐free survival (PFS) after androgen deprivation therapy (ADT) in patients in the recurrent/metastatic (R/M) cohort for AR pathway activity score (*p* = 0.13) (*a*) and SRD5A1 expression (*p* = 0.008) (*b*). Disease‐free survival (DFS) after adjuvant ADT in patients in the locally advanced (LA) cohort for AR pathway activity (*p* = 0.061) (*c*) and SRD5A1 expression (*p* = 0.73) (*d*). [Color figure can be viewed at http://wileyonlinelibrary.com]

Patients with clinical benefit in the R/M cohort had a significantly higher AR pathway activity score compared to those without. A ROC‐curve of the AR pathway activity score to predict clinical benefit in the R/M cohort was constructed (Fig. 2 *a*). The area under the curve was 0.75 (95% CI 0.54–0.95, *p* = 0.035). The optimal cut‐off value for predicting clinical benefit was an AR pathway activity score of 52.9, which resulted in a sensitivity of 55.6%, a specificity of 95.2%, a positive predictive value of 83.3% and a negative predictive value of 83.3%. Using this cut‐off value, Kaplan–Meier survival curves were constructed for PFS (Fig. 3 *a*) and OS (Supporting Information Fig. S3 *a*) in the R/M cohort. The median PFS after treatment with palliative ADT was 2.9 months (95% CI 2.7–3.1 months) for patients with an inactive AR pathway and 9.9 months (95% CI 1.5–18.3 months) for patients with an active AR pathway (*p* = 0.13). The median OS was 25.9 months (95% CI 8.9–42.9 months) for patients with an inactive AR pathway and 43.6 months (95% CI 0.9–86.3 months) for patients with an active AR pathway (*p* = 0.39). Subsequently, this cut‐off value was applied to the LA cohort and the median DFS was 17.7 months (95% CI 8.4–27.0 months) for patients with an inactive AR pathway and 22.8 months (95% CI could not be calculated because only one patient had a recurrence) for patients with an active AR pathway (*p* = 0.061; Fig. 3 *c*). In the LA cohort, differences in OS were not calculated because of insufficient follow‐up.

### Prediction of clinical benefit from ADT using SRD5A1 mRNA expression

A significant correlation between *SRD5A1* mRNA expression of the primary tumor tissue and clinical benefit was found. The area under the ROC curve for *SRD5A1* in the R/M cohort (Fig. 2 *b*) was 0.79 (95% CI 0.62–0.96, *p* = 0.014). The optimal cut‐off value for predicting clinical benefit was an *SRD5A1* expression of 2.75, which resulted in a sensitivity of 77.8%, a specificity of 75.0%, a positive predictive value of 58.3% and a negative predictive value of 88.2%. Using this cut‐off value, Kaplan–Meier survival curves were constructed for PFS (Fig. 3 *b*) and OS (Supporting Information Fig. S3 *b*) in the R/M cohort. The median PFS after treatment with palliative ADT was 2.8 months (95% CI 2.3–3.3 months) for patients with a low *SRD5A1* expression and 5.6 months (95% CI 0.0–13.2 months) for patients with a high *SRD5A1* expression (*p* = 0.008). The median OS was 24.2 months (95% CI 2.9–45.5 months) for patients with a low *SRD5A1* expression and 46.3 months (95% CI 0.0–92.8 months) for patients with a high *SRD5A1* expression (*p* = 0.069). Subsequently, this cut‐off value was applied to the LA cohort and the median DFS was 17.7 months (95% CI could not be calculated) for patients with a low *SRD5A1* expression and 22.8 months (95% CI 6.3–39.3 months) for patients with a high *SRD5A1* expression (*p* = 0.73; Fig. 3 *d*). In the LA cohort, differences in OS were not calculated because of insufficient follow‐up.

### Combining the AR pathway activity score and SRD5A1 expression

**Figure 4:**
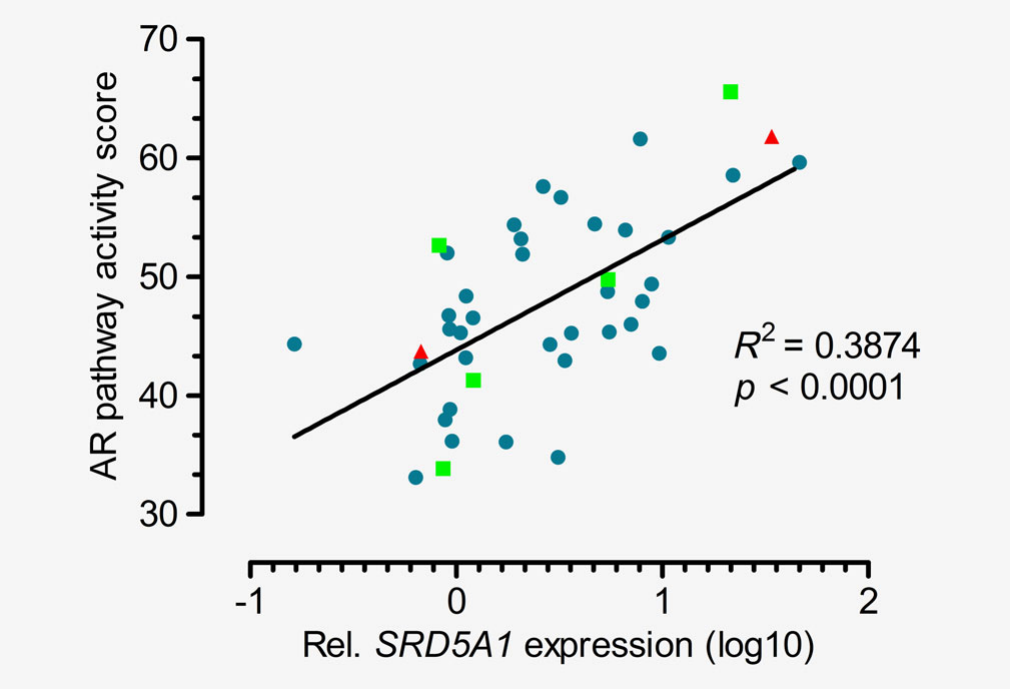
Correlation of relative SRD5A1 expression levels (normalized to HPRT1 housekeeping gene levels) and AR pathway activity scores measured in primary salivary duct carcinomas (blue dots, *n* = 36), regional lymph node metastases (green squares, *n* = 5) and distant metastases (red triangle, *n* = 2) of patients in the recurrent/metastatic cohort and locally advanced cohort. R‐squared and *p* values of the linear regression analysis are shown. [Color figure can be viewed at http://wileyonlinelibrary.com]

Kaplan–Meier curves were constructed for PFS, OS (R/M cohort) and DFS (LA cohort) for patients with a high AR pathway activity score (>52.9) and high *SRD5A1* expression (>2.75) *vs*. negative cases for both factors. Discrepant cases were left out. This resulted in a median PFS of 10.6 months (95% CI 3.1–18.1 months) and 2.8 months (95% CI 2.2–3.4 months, *p* = 0.005), a median OS of 46.3 months (95% CI 9.6–83.0 months) and 24.2 months (95% CI 20.7–27.7 months, *p* = 0.11), and a median DFS of 22.8 months (95% CI could not be calculated) and 17.7 months (95% CI could not be calculated, *p* = 0.32), respectively. Furthermore, a significant correlation between the AR pathway activity score and *SRD5A1* expression factors was found (*R* ^2^ = 0.39, *p* < 0.001; Fig. 4).

### Prediction of clinical benefit in primary tumor *vs*. metastatic tissue

In the R/M cohort, primary tumor tissue was used in 23 patients, and tissue for regional lymph node metastases and distant metastases prior to ADT in 4 and 3 patients, respectively. *SRD5A1* expression levels and AR pathway activity scores were not significantly different between primary tumor tissue, lymph node metastatic tissue and distant metastatic tissue (Supporting Information Fig. S4). However, the difference in *SRD5A1* expression levels and AR pathway activity scores between patients with and without clinical benefit remained significant when the analyses were performed in metastatic tissue only (*n* = 7; *SRD5A1* expression 27.73 (IQR 21.5– ‐) *vs*. 0.85 (IQR 0.71–1.13), *p* < 0.001 and median AR pathway activity 63.7 (IQR 61.8 – ‐) *vs*. 43.7 (IQR 37.6–51.5), *p* = 0.019, respectively. Supporting Information Fig. S5). Both ROC‐curves showed an area under the curve of 1 (95% CI 1–1, *p* = 0.064 and 95% CI 1–1, *p* = 0.053, respectively, Supporting Information Fig. S6). Median PFS was significantly longer for patients with high *SRD5A1* expression levels (2.5 *vs*. 10.6 months, *p* = 0.049) and high AR pathway activity scores (2.5 *vs*. 10.6 months, *p* = 0.041). Median OS was not different for both factors. For primary tumor tissue (*n* = 23), the *SRD5A1* expression levels and AR pathway activity scores were higher in patients with clinical benefit, but significance was lost (*p* = 0.57 and *p* = 0.20, respectively).

### HER2 assessment

HER2 gene (*ERBB2*) amplification was determined in all patients in the R/M cohort and 10 of 14 patients in the LA cohort. Of these 40 patients, 14 patients (35.0%) had a tumor with an *ERBB2* amplification (Tables 1 and 2). In the R/M cohort, no significant differences were found between the clinical benefit rate in patients with or without *ERBB2* amplification (20.0% *vs*. 31.6%, *p* = 0.68) or OS (median 40.4 months, 95% CI 31.1–49.7 months *vs*. 22.4 months, 95% CI 17.6–27.2 months, *p* = 0.26).

### Mutation analysis

Mutation analysis was performed in all 30 patients in the R/M cohort. In 12 tumors (40.0%), no driver mutations were found. In the other 18 tumors, mutations were detected in the *TP53* (*n* = 11), *PIK3CA* (*n* = 5), *HRAS* (*n* = 4), *PTEN* (*n* = 2), *BRAF* (*n* = 2), *AKT1* (*n* = 1) and *ERBB2* (*n* = 2) genes (Table 1 and Supporting Information Table S3). In the R/M cohort, no significant differences were found between the clinical benefit rate in patients with or without the presence of one of these gene mutations (33.3% *vs*. 25.0%, *p* = 0.70) or OS (24.2 months, 95% CI 19.1–29.3 months *vs*. 43.6 months, 95% CI 36.5–50.7 months, *p* = 0.39).

### Other palliative systemic treatments

Besides first‐line ADT, patients in the R/M cohort received other palliative systemic treatments after PD on ADT. An overview of the treatments is listed in Supporting Information Table S4. Based on the AR pathway activity score, patients with an inactive AR pathway received a mean number of 1.9 palliative systemic treatments *vs*. 1.5 in patients with an active AR pathway. Patients with an inactive AR pathway more often received second‐line ADT (37.5% *vs*. 16.7%), third‐line ADT (8.3% *vs*. 0.0%), chemo and/or targeted therapy (37.5% *vs*. 33.3%) and immunotherapy (4.2% *vs*. 0.0%).

## Discussion

In our study, potential mechanisms of primary ADT resistance in SDC patients were investigated in order to predict clinical benefit from ADT. We are the first to describe that *SRD5A1* expression levels and AR pathway activity scores were significantly higher in patients with clinical benefit compared to those without. Survival analysis in the R/M cohort revealed that median PFS was longer for patients with high *SRD5A1* expression levels (2.8 *vs*. 5.6 months) and high AR pathway activity scores (2.9 *vs*. 9.9 months). Differences in OS were not found, but many patients received additional palliative systemic treatments, follow‐up was relatively short and the cohorts were relatively small. The threshold for elevated *SRD5A1* expression and for high AR pathway activity was set (discovered) on the R/M cohort, and subsequently evaluated for differences in DFS in an independent cohort of locally advanced SDC patients receiving adjuvant ADT (*n* = 14). For the AR pathway activity analysis and not *SRD5A1* expression, a trend toward a better DFS was found. Therefore, the AR pathway activity score is most promising as predictive factor for clinical benefit from ADT, but validation in a prospective study is needed before this method can be put into daily practice. Furthermore, it would be interesting to evaluate the prognostic value of AR‐pathway activity and *SRD5A1* expression, as the survival differences may also be a result of a more indolent (well‐differentiated) tumor.

The predictive value of the AR pathway analysis may be further improved by using metastatic tissue only. In our study, only 7 of 30 samples in the R/M cohort were obtained from regional lymph node metastases or distant metastases prior to treatment. Nonetheless, the differences in *SRD5A1* expression levels and AR pathway activity scores were still significant between patient with and without clinical benefit.

Combining *SRD5A1* expression and AR pathway activity score showed a modest improvement in the prediction of clinical benefit in the R/M cohort, but worse prediction compared to the AR pathway activity score alone in the LA cohort. The most logical explanation for the lack of added value is that both biomarkers are either directly (the AR pathway activity score) or indirectly (*SRD5A1* gene expression) associated with activity of the AR pathway, as SRD5A1 overexpression results in increased production of androgens in prostate cancer,^23^ resulting in AR pathway activation. Although intratumoral androgen synthesis has not yet been demonstrated in SDC, the former also may be an explanation for the positive correlation between *SRD5A1* expression levels and AR pathway activity in SDC. More importantly, the level of *SRD5A1* expression may have therapeutical consequences, as ADT response in *SRD5A1* overexpressing tumors may be further enhanced by adding the SRD5A1 and SRD5A2 inhibitor dutasteride to the ADT regimen. In prostate cancer models, it was shown that dutasteride synergistically suppresses tumor cell proliferation when combined with enzalutamide (a new generation AR antagonist that is more potent than bicalutamide).^24^ Therefore, combining dutasteride with ADT is a rational approach for future clinical trials in AR‐positive SDC, especially in a translational setting in which the use of *SRD5A1* expression levels and/or AR pathway activity scores as predictive biomarkers are confirmed, preferably on metastatic tissue.

The other potential primary resistance mechanisms were not significantly different between patients with and without clinical benefits from ADT and therefore cannot be used as predictive factors. However, some noteworthy findings were done. Although AR protein expression was in general high, one tumor lacked AR protein expression. This patient started palliative ADT based on an AR‐positive lung metastasis but had an AR negative epidural metastasis. This case shows the possible heterogeneity in AR expression in SDC metastases and could explain treatment resistance in such cases. Furthermore, we detected *AR‐V7* mRNA expression in 95.3% of primary tumor samples, which is more frequent than the 37–70% of *AR‐V7* positive SDC cases reported earlier.^12^, ^15^, ^25^ In CRPC, AR‐V7 expression explains at least in part the resistance to ADT,^26^ and *AR‐V7* expression is elevated in response to ADT.^14^ We found *AR‐V7* expression prior to ADT. Whether expression levels of *AR* or *AR‐V7*, or other genes, change during ADT in SDC remains to be investigated. Finally, genetic alterations such as *ERBB2* amplification and functionally relevant mutations in oncogenes and tumor suppressor genes were found in 35.0 and 60.0% of patients, respectively, which is in accordance with the literature.^12^ This may cause ADT resistance by activating other tumor‐driving pathways, such as the PI3K‐AKT pathway, in the presence of an active AR pathway. It is therefore interesting to note that a positive HER2 status did not affect clinical benefit from ADT in AR and HER2 positive SDC patients, which is in line with data from other research groups.^6^, ^7^ Therefore, ADT seems to be a good treatment option in these patients, but the presence of other targetable genetic markers stresses the importance of prediction of clinical benefit in order to select the most suitable therapy. In our study, HER2 status was tested with FISH as first test according to ASCO‐CAP guideline.^20^ Because immunohistochemistry was only added in case of inconclusive results, we might have missed an occasional immunohistochemically 3+ case. In the most recent ASCO‐CAP guideline for HER2 testing in breast cancer, this has been addressed and the guideline has been adjusted accordingly.^27^

The positive results in our study are remarkable since our study has several limitations. First, the cohorts were relatively small, which is typical for a rare cancer but troublesome for elucidating predictive factors for clinical benefit. Second, not all patients received the same ADT, and compliance to ADT may differ, all of which could influence ADT response independent of AR pathway activity scores. Furthermore, for most patients, prediction of clinical benefit was based on primary tumor tissue samples, while treatment was installed when patients developed metastatic disease, with a varying time interval between primary tumor resection and development of metastases. It is well known that molecular characteristics frequently vary between primary tumors and metastases.^18^ For breast cancer, it was already shown that signaling pathway activity scores vary to a large extent between primary and distant metastases.^28^ In our study, the number of metastatic tissue samples, although small, was sufficient to detect significant differences between patients with and without clinical benefit. Finally, the quality of the tumor specimens varied. Some of the FFPE tumor specimens were old, up to more than 16 years, and the tumor percentage was low in some tumor specimens, resulting in “contamination” with benign salivary epithelial cells. Nonetheless, these tumor specimens endured RNA quality control and could still be used our analyses.

In conclusion, in our study, potential intrinsic ADT resistance mechanisms were studied in AR‐positive SDC patients that received ADT. We are the first to describe low *SRD5A1* expression and low AR pathway activity scores as potential primary ADT resistance mechanisms in SDC patients. We speculate, based on our findings and studies in prostate cancer, that ADT response in *SRD5A1* overexpressing tumors may be further enhanced by adding the SRD5A1 inhibitor dutasteride to the ADT regimen. The AR pathway activity score is a promising predictive biomarker for clinical benefit from ADT in SDC. Validation in a prospective study is needed, preferably in metastatic tissue samples.

## Supporting information


**Appendix S1**: Supporting Information

## Data Availability

The data that support the findings of our study are available from the corresponding author upon reasonable request.
