# Supplementary material for: Prediction of clinical benefit from androgen deprivation therapy in salivary duct carcinoma patients
Source: Int J Cancer. 2019 Dec 12;146(11):3196–206. doi: 10.1002/ijc.32795 (PMC7187215; doi:10.1002/ijc.32795)
Supplement: Supplementary file 1 — Appendix S1: Supporting Information [file IJC-146-3196-s001.pdf]

Supplementary data

Prediction of clinical benefit from androgen deprivation therapy in salivary duct carcinoma patients

Authors

Wim van Boxtel, Gerald W. Verhaegh, Ilse A. van Engen - van Grunsven, Dianne van Strijp, Leonie I. Kroeze, Marjolein J. Ligtenberg, Hans B. van Zon, Yara Hendriksen, Diederick Keizer, Anja van de Stolpe, Jack A. Schalken, Carla M. van Herpen

Table of contents

Supplementary tables
1: Sequences and amplicon sizes of primer pairs used for qPCR analysis 2
2: Target genes in 29-gene panel for smMIP analysis 2
3: Specification of DNA mutations and allele frequencies of patients in the R/M cohort 3
4: Overview of palliative systemic treatments of patients in the R/M cohort 4

Supplementary figures
1: Box plots of ADT primary resistance mechanisms 5
2: Correlation of relative AR and AR-V7 expression levels 6
3: Kaplan-Meier overall survival curves after ADT in the R/M cohort 7
4: Box plots of relative SRD5A1 gene expression levels and AR pathway activity scores 7
5: Box plots of relative SRD5A1 gene expression levels and AR pathway activity scores in metastatic tissue only 7
6: ROC-curves to predict clinical benefit from ADT by using metastatic tissue only 8

Supplementary table 1: Sequences and amplicon sizes of primer pairs used for qPCR analysis.

Gene	Forward primer 5’ → 3’	Reverse primer 5’ → 3’	Amplicon size (bps)
AR (full-length)	TACCAGCTCACCAAGCTCCT	CAGGTCAAAAGTGAACTGATGC	72
AR-V7	CGTCTTCGGAAATGTTATGAAGC	TGCAATTGCCAACCCGGAAT	64
AKR1C3	CCAGGACTCAAGTACAAGCCT	TCTAGCAATTTACTCCGGTTGA	74
CYP17A1	AAGGGCAAGGACTTCTCTGG	ACCCTTACGGTTGTTGGACG	69
SRD5A1	AGGAATCTCAGAAAACCAGGAGA	GTTGGCTGCAGTTACGTATTCA	78
SRD5A2	CCCTGATGGGTGGTACACAG	TGAATGTTTATTCCCATTCCCAAA	78
HPRT1	CTGGAAAGAATGTCTTGATTGTGG	GCCTGACCAAGGAAAGCAAAG	78

Supplementary table 2: Target genes in 29-gene panel for smMIP analysis.

**Gene**	**NCBI Reference Sequence Database**	**Region of interest**
AKT1	NM_005163.2	Codon 17
AKT2	NM_001626.5	Codon 17
AKT3	NM_181690.2	Codon 17
ALK	NM_004304.4	Codons 1059-1150, 1173-1278
ARAF	NM_001654.4	Codon 214
BRAF	NM_004333.4	Codons 455-488, 566-580, 594-605
DDR2	NM_006182.2	Codons 503-856
EGFR	NM_005228.4	Codons 434-499, 688-875
ERBB2	NM_004448.3	Codons 310, 650-883
GNA11	NM_002067.4	Codons 183 and 209
GNAQ	NM_002072.4	Codons 183 and 209
GNAS	NM_000516.5	Codons 201 and 227
HRAS	NM_005343.3	Codons 12, 13, 59 and 61
IDH1	NM_005896.3	Codon 132
IDH2	NM_002168.3	Codons 140 and 172
JAK2	NM_004972.3	Codon 617
KIT	NM_000222.2	Codons 412-513, 550-591, 640-787, 799-850
KRAS	NM_004985.4	Codons 12, 13, 59, 61, 117 and 146
MAP2K1	NM_002755.3	Codons 28-231
MET	NM_001127500.2	Codons 168, 375, 982-1027, 1230-1284, 1304
MTOR	NM_004958.3	Codons 1458-1489, 1789-1820, 1971-1995, 2194-2220, 2404-2433, 2484-2509
NRAS	NM_002524.4	Codons 12, 13, 59, 61, 117 and 146
PDGFRA	NM_006206.5	Codons 552-595, 632-667, 824-848
PIK3CA	NM_006218.3	Codons 345, 420, 539-554, 1043-1050
POLE	NM_006231.3	Codons 268-491
PTEN	NM_000314.6	Codons 86-267, 276-342
RAF1	NM_002880.3	Codons 257-261
ROS1	NM_002944.2	Codons 1927-2189
TP53	NM_000546.5	>95% of the coding sequences and splice sites (-5/+5)

Supplementary table 3: Specification of DNA mutations and allele frequencies of patients in the recurrent/metastatic cohort.

Patient no.	Driver mutations	Allele frequency
1	None	-
2	TP53: c.587G>A (p.(Arg196Gln))	6%
3	TP53: c.549_558del (p.(Asp184fs))	22%
4	ERBB2: c.2263_2264delinsCC (p.(Leu755Pro))	42%
	TP53: TP53 c.626_627del (p.(Arg209fs))	24%
5	PTEN c.528T>G (p.(Tyr176*))	55%
	TP53 c.1024C>T (p.(Arg342*))	28%
6	TP53 c.854_855del (p.(Glu285fs))	43%
	PTEN c.569_570dup (p.(Val191fs))	21%
7	None	-
8	TP53 c.892G>T (p.(Glu298*))	53%
9	None	-
10	None	-
11	HRAS: c.181C>A (p.(Gln61Lys))	17%
	PIK3CA: c.3140A>G (p.(His1047Arg))	23%
12	AKT1: c.49G>A (p.(Glu17Lys))	17%
	BRAF: c.1799T>A (p.(Val600Glu)) alias p.V600E	24%
13	ERBB2: c.2264T>C (p.(Leu755Ser))	26%
	TP53: c.1000G>T(p.(Gly334Trp));	24%
14	PIK3CA: c.1633G>A (p.(Glu545Lys))	15%
	HRAS c.182A>G (p.(Gln61Arg))	23%
15	None	-
16	None	-
17	None	-
18	BRAF: c.1799T>A (p.(Val600Glu)) alias p.V600E	39%
19	None	-
20	None	-
21[#]	TP53: c.578A>G (P.(His193Arg))	29%
22	TP53: c.370del (p.(Cys124fs))	14%
23	TP53: c.949C>T (p.(Gln317*))	29%
24	TP53: c.626_627del (p.Arg209Lysfs*6))	30%
25	None	-
26	PIK3CA: c.3140A>T (p.(His1047Leu))	22%
27	None	-
28	HRAS: HRAS: c.181C>A (p.(Gln61Lys))	28%
	PIK3CA: c.1633G>A (p.(Glu545Lys))	20%
	PIK3CA: c.3140A>G (p.(His1047Arg))	17%
29	None	-
30	PIK3CA c.3140A>G (p.(His1047Arg))	16%
	HRAS c.182A>G (p.(Gln61Arg))	32%

#, Because of low DNA yield other mutations could have been missed.

Supplementary table 4: Overview of palliative systemic treatments of patients in the recurrent/metastatic (R/M) cohort

	Patients with an inactive AR pathway (*n*=24)<br>No. of patients (%)	Patients with an active AR pathway (*n*=6)<br>No. of patients (%)
1st-line ADT		
• Bicalutamide 150 mg OD	19 (79.2%)	4 (66.7%)
• LHRH-analog plus bicalutamide 50 mg OD	5 (20.8%)	2 (33.3%)
2nd-line ADT		
• LHRH-analog plus bicalutamide 50 mg OD	7 (29.2%)	1 (16.7%)
• LHRH-analog	2 (8.3%)	0 (0.0%)
3rd-line ADT		
• LHRH-analog plus enzalutamide 160 mg OD	2 (8.3%)	0 (0.0%)
1st-line chemo and/or targeted therapy		
• Docetaxel	3 (12.5%)	0 (0.0%)
• Docetaxel plus trastuzumab plus pertuzumab	2 (8.3%)	1 (16.7%)
• Trastuzumab plus pertuzumab	0 (0.0%)	1 (16.7%)
• Carboplatin plus paclitaxel	1 (4.2%)	0 (0.0%)
• Cyclophosphamide plus doxorubicin plus cisplatin	1 (4.2%)	0 (0.0%)
• Pembrolizumab	1 (4.2%)	0 (0.0%)
• Vemurafenib plus cobimetinib	1 (4.2%)	0 (0.0%)
2nd-line chemo and/or targeted therapy		
• Trastuzumab-emtansin	1 (4.2%)	0 (0.0%)

ADT: androgen deprivation therapy, OD: once daily, AR: androgen receptor.

Supplementary figure 1: Box plots of androgen deprivation therapy (ADT) primary resistance mechanisms in patients with recurrent/metastatic salivary duct carcinoma with and without clinical benefit from ADT. **A**: *AR* gene expression levels. **B**: androgen receptor splice variant 7 (*AR-V7*) gene expression levels. **C**: Androgen receptor (AR) protein expression levels. AR expression was scored considering the staining intensity (0=negative, 1=weak, 2=moderate, 3=strong) and the percentage of positive nuclei (0=<10%, 1=10-30%, 2=30-70%, 3=>70%). The final staining score was recorded as the sum of the staining intensity and the staining extent.[10] **D**: Androgen receptor (AR) pathway activity scores. **E**: *Aldo-keto reductase family 1 member C3 (AKR1C3)* gene expression levels. **F**: *Steroid 5 alpha-reductase 1 (SRD5A1)* gene expression levels. **G**: *SRD5A2* gene expression levels. All gene expression levels were normalized to *hypoxanthine phosphoribosyltransferase 1 (HPRT1)* housekeeping gene levels. Progressive disease at first evaluation or stable disease <6 months was categorized as no clinical benefit, and complete remission, partial response or stable disease for >6 months was defined as clinical benefit, both according to RECIST criteria.

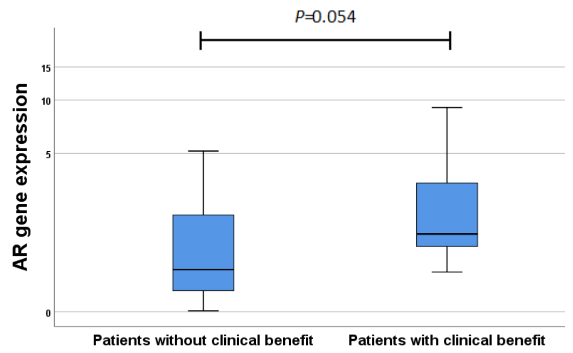

Supplementary figure 1a

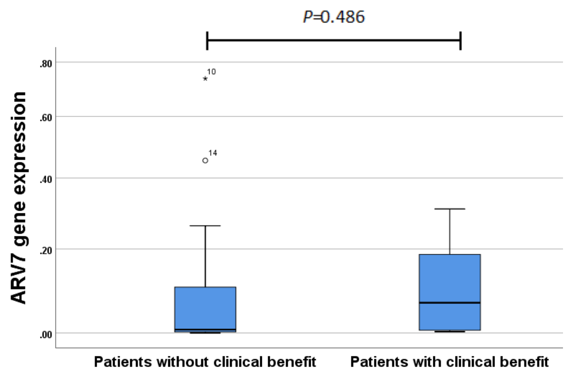

Supplementary figure 1b

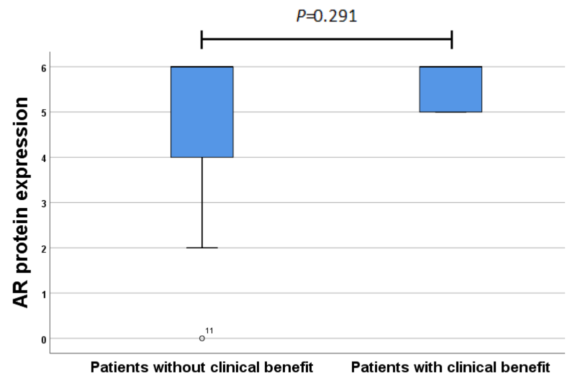

Supplementary figure 1c

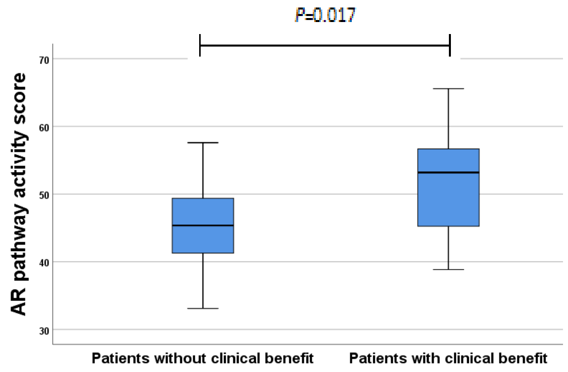

Supplementary figure 1d

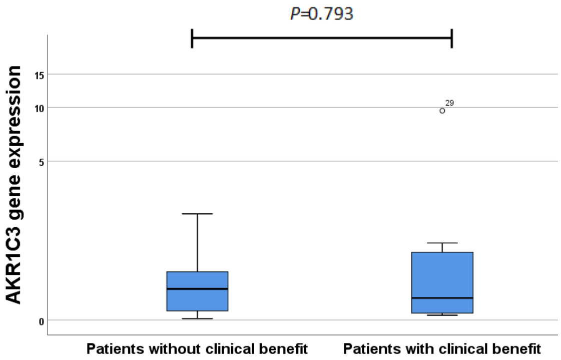

Supplementary figure 1e

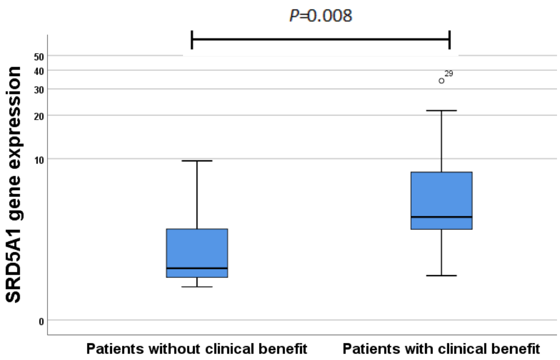

Supplementary figure 1f

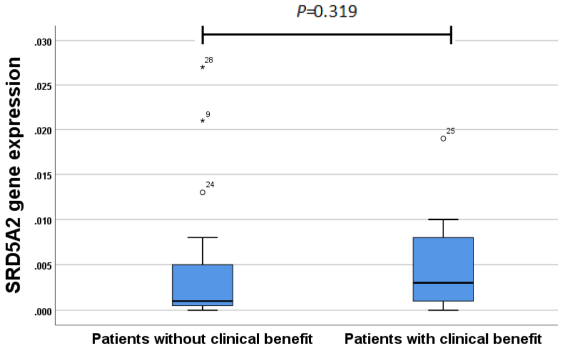

Supplementary figure 1g

Supplementary figure 2: Correlation of relative *AR* and *AR-V7* expression levels (normalized to *HPRT1* housekeeping gene levels) measured in primary salivary duct carcinomas (in blue, *n*=36), regional lymph node metastases (in green, *n*=5) and distant metastases (in red, *n*=2) of patients in the recurrent/metastatic cohort and locally advanced cohort. R-squared and p-values of the linear regression analysis are shown.

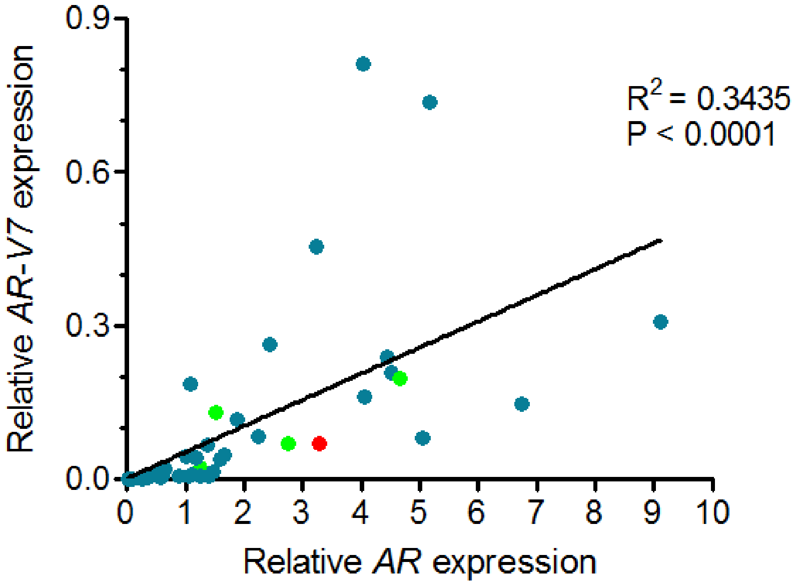

Supplementary figure 3: Kaplan-Meier overall survival (OS) curves after androgen deprivation therapy (ADT) in patients in the recurrent/metastatic (R/M) cohort for AR pathway activity score (a) and *SRD5A1* expression (b).

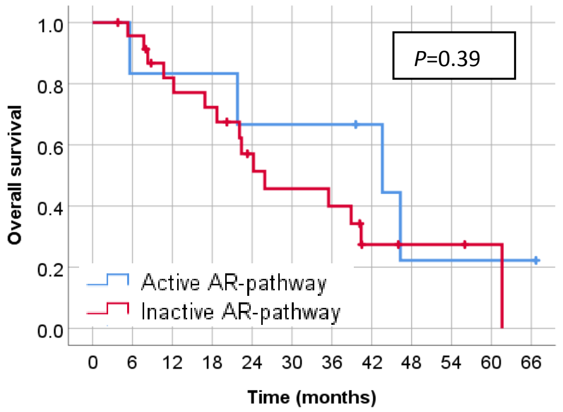

Supplementary figure 3a

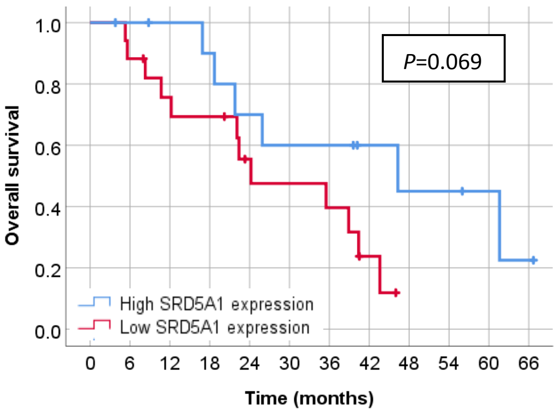

Supplementary figure 3b

Supplementary figure 4: Box plots of relative *SRD5A1* gene expression levels (a) and AR pathway activity scores (b) in the recurrent/metastatic cohort. T, primary SDC tumor (*n*=23); N, lymph node metastasis (*n*=4); M, distant metastasis (*n*=3).

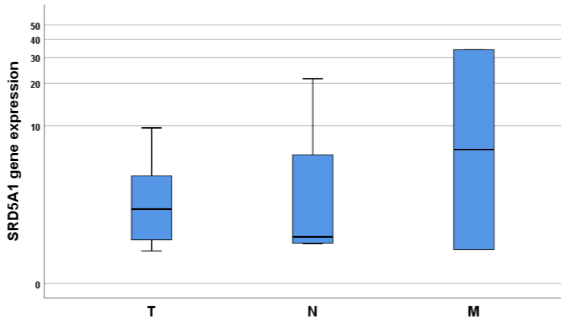

Supplementary figure 4a

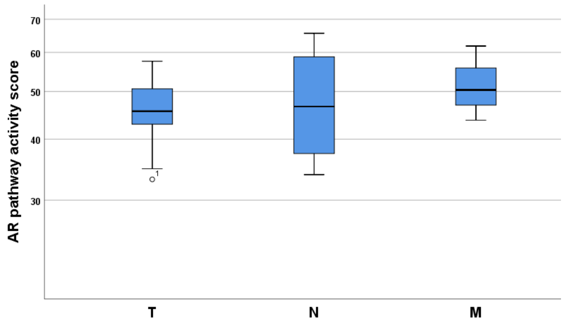

Supplementary figure 4b

Supplementary figure 5: Box plots of relative *SRD5A1* gene expression levels (a) and AR pathway activity scores (b) in metastatic tissue (*n*=7) of patients with recurrent/metastatic salivary duct carcinoma with and without clinical benefit from ADT.

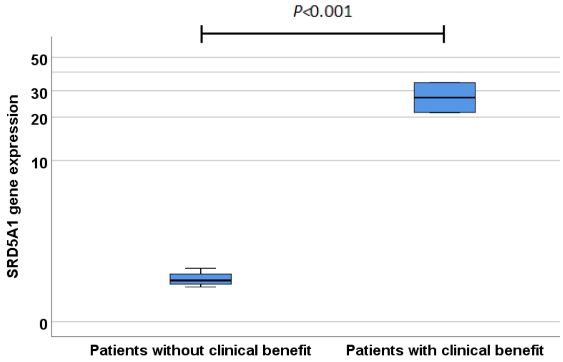

Supplementary figure 5a

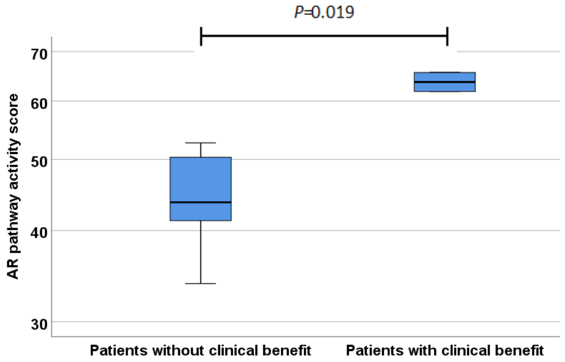

Supplementary figure 5b

Supplementary figure 6: Receiver operating characteristic (ROC)-curves describing the sensitivity and specificity to predict clinical benefit from androgen deprivation treatment by using metastatic tissue only in the R/M cohort (*n*=7). a: ROC-curve of androgen receptor pathway analysis. A cut-off value of 57.2 was used for the subsequent survival analyses, which has a sensitivity of 1.000 and 1-specificity of 0.000 in this cohort. b: ROC-curve of *steroid 5 alpha-reductase 1 (SRD5A1)* gene expression levels. A cut-off value of 11.34 was used, which has a sensitivity of 1.000 and 1-specificity of 0.000 in this cohort. AUC: area under the curve. CI: confidence interval.

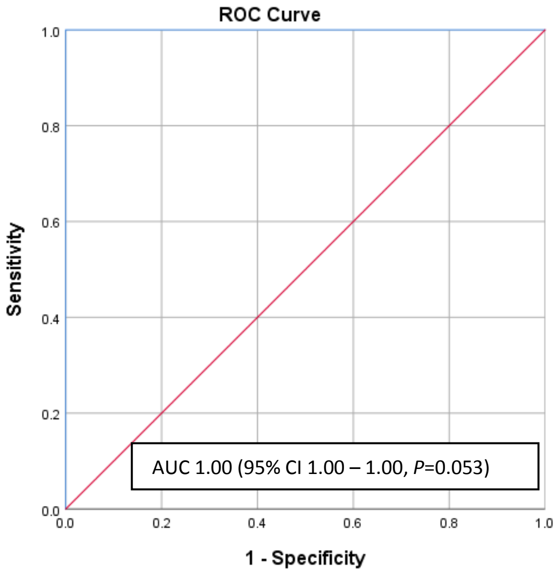

Supplementary figure 6a

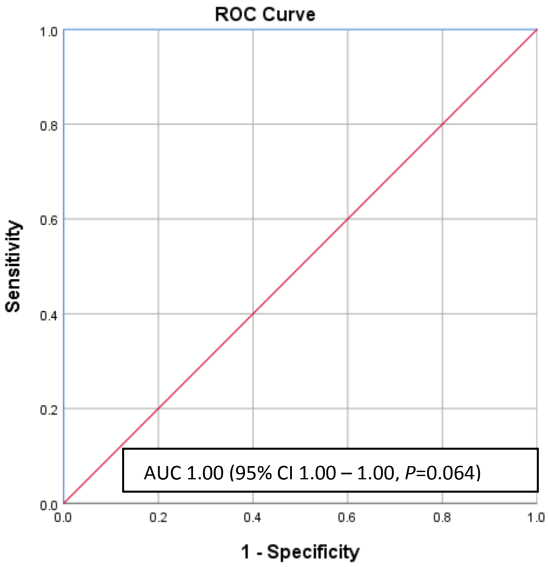

Supplementary figure 6b
